# Safety and efficacy of tislelizumab plus chemotherapy versus chemotherapy alone as neoadjuvant treatment for patients with locally advanced gastric cancer: real-world experience with a consecutive patient cohort

**DOI:** 10.3389/fimmu.2023.1122121

**Published:** 2023-05-04

**Authors:** Qi Jiang, Weizhen Liu, Xiangyu Zeng, Chenggang Zhang, Yuqiang Du, Liwu Zeng, Yuping Yin, Jun Fan, Ming Yang, Kaixiong Tao, Peng Zhang

**Affiliations:** ^1^ Department of Gastrointestinal Surgery, Union Hospital, Tongji Medical College, Huazhong University of Science and Technology, Wuhan, Hubei, China; ^2^ Department of Pathology, Union Hospital, Tongji Medical College, Huazhong University of Science and Technology, Wuhan, Hubei, China

**Keywords:** neoadjuvant chemotherapy, gastric cancer, tislelizumab, gastrectomy, efficacy

## Abstract

**Objectives:**

Immunotherapy plus chemotherapy has recently been applied in the neoadjuvant treatment for locally advanced gastric cancer (LAGC), while its superiority over neoadjuvant chemotherapy (NACT) alone remains to be explored. This study explored the safety and efficacy of NACT plus tislelizumab in patients with LAGC.

**Methods:**

The data on patients with LAGC who received NACT combined with radical gastrectomy and NACT plus tislelizumab followed by radical gastrectomy was retrospectively collected. Clinicopathological characteristics of the two groups were compared.

**Results:**

A total of 119 and 50 patients with gastric cancer treated with NACT and NACT plus tislelizumab, respectively, were enrolled. No significant difference was found between the baseline data of the two groups. The operative time (210.5 ± 70.4 min *vs.* 237.6 ± 68.4 min, *P*=0.732), intraoperative blood loss (157.8 ± 75.9 ml *vs.* 149.1 ± 92.5 ml, *P*=0.609), and number of dissected lymph nodes (24.7 ± 9.3 *vs.* 28.1 ± 10.3, *P*=0.195) was not statistically different between the two groups. In comparison to the NACT plus tislelizumab group, the R0 resection rate (100% *vs.* 89.9%, *P*=0.019) and pathologic complete response rate (26.0% *vs.* 3.4%, *P*<0.001) were significantly lower in the NACT group. The postoperative complication rates were 24.4% and 26.0% in the NACT and NACT plus tislelizumab groups with no significant difference *(P*=0.823). In subgroup analysis, tumor regression grade (TRG) (TRG 3: 72.3% *vs.* 23.5%, *P*<0.001) and ypN stage (stages 2–3: 46.8% *vs.* 5.9%, *P*=0.003) in the NACT group were significantly higher compared with the NACT plus tislelizumab group in esophagogastric junction carcinoma.

**Conclusion:**

Compared with the S-1 and oxaliplatin (SOX) or 5-fluorouracil, folinic acid, and oxaliplatin (FOLFOX) NACT regimen, NACT plus tislelizumab significantly improved the efficacy and R0 resection rate of LAGC without increasing the incidence of perioperative complications, particularly in esophagogastric junction carcinoma.

## Introduction

Gastric cancer is among the most prevalent malignancies and one of the leading causes of cancer-related mortality. About 1.08 million new gastric cancer cases and 760,000 gastric cancer-related deaths are reported worldwide annually (1). Most Chinese patients with gastric cancer (70%) are in locally advanced stage with poor prognosis when diagnosed (2). Recently, a range of large prospective studies have shown that neoadjuvant chemotherapy (NACT) followed by radical gastrectomy significantly improves progression-free survival (PFS) and overall survival (OS) in patients with locally advanced gastric cancer (LAGC). Therefore, NACT combined with radical gastrectomy has become a standard treatment for LAGC (3–6). However, approximately 40% of gastric cancer patients treated with NACT experience recurrence and metastasis within 3 years after surgery (3–5, 7, 8). Therefore, exploring new models of neoadjuvant therapy for LAGC is essential.

Immune checkpoint inhibitors targeting programmed death 1 (PD-1) protein or PD-1 ligand 1 usher in a new era of oncology treatment. Initially, immunotherapy was investigated in the third and second line treatment of advanced gastric cancer. Studies including KEYNOTE-059, ATTRACTION-2, KEYNOTE-061, and CheckMate-032 demonstrated that immunotherapy significantly improved the prognosis of such patients compared to chemotherapy (9–12). Based on these exciting results, Studies including KEYNOTE-062, ATTRACTION-4, and CheckMate-649 began to include the patients with advanced gastric cancer treated in first line and confirmed that PD-1 antibody combined with chemotherapy can significantly improve PFS and OS in unresectable advanced or metastatic gastric cancer patients, and this treatment has become a first-line alternative for such patients (6, 13–15).

Subsequently, a series of prospective studies, including our previous study, explored the value of neoadjuvant immunotherapy in gastric cancer and found that NACT plus immunotherapy enables patients with LAGC to achieve high pathologic complete response (pCR) and major pathologic response rates (16–18). However, most of them are single-arm studies with limited strength of evidence. The abstract reported by Li et al. in ASCO 2022 showed that preoperative immunotherapy plus chemotherapy/radiotherapy for resectable gastric cancer (stage II-IV) had better pathologic responses and R0 resection rates than chemotherapy (19). Lin et al. and Su et al. compared the efficacy of preoperative immunotherapy plus chemotherapy and chemotherapy alone in gastric cancer with serosal invasion and advanced gastric cancer, respectively, and found that preoperative chemotherapy plus immunotherapy resulted in a better pathological regression rate of the tumor (20, 21). The above studies have important implications for grasping the value of preoperative chemotherapy plus immunotherapy in gastric cancer, but they all included a considerable number of advanced gastric cancer cases. It remains to be investigated whether NACT combined with immunotherapy is superior to NACT alone in LAGC patients. This study aimed to explore the safety and efficacy of NACT plus tislelizumab by comparing data of LAGC patients treated with NACT or NACT plus tislelizumab over a decade to provide a reference for clinicians during treatment planning for such patients.

## Materials and methods

### Patients

This study retrospectively collected data on patients with LAGC who received NACT combined with radical gastrectomy at the Union Hospital of Tongji Medical College, Huazhong University of Science and Technology, between January 2012 and September 2021 and those who underwent NACT plus tislelizumab followed by radical gastrectomy between October 2021 and August 2022. Patients aged 18 to 80 years old with cT3-4aNanyM0 esophagogastric junction carcinoma or cT1-4aN+M0 non-esophagogastric junction carcinoma, who were evaluated using endoscopic ultrasonography and computed tomography (CT), with Her2(-) expression assessed using immunohistochemistry, with Eastern Cooperative Oncology Group score of 0–2, and who received two to four cycles of S-1 and oxaliplatin (SOX) or 5-fluorouracil, folinic acid, and oxaliplatin (FOLFOX) NACT regimen or NACT plus tislelizumab were included in the study. Patients with distant metastasis, with gastric stump cancer or other associated malignant tumors, who received neoadjuvant therapies other than chemotherapy or chemotherapy combined with immunotherapy, such as neoadjuvant targeted therapy and neoadjuvant chemoradiotherapy, and with incomplete clinicopathological data were excluded. All patients received NACT plus tislelizumab in a phase II clinical trial (NCT04890392) at our center. This study was authorized by the Institutional Review Board of the Union Hospital of Tongji Medical College and performed in compliance with the Declaration of Helsinki.

### Treatments

**Figure 1:**
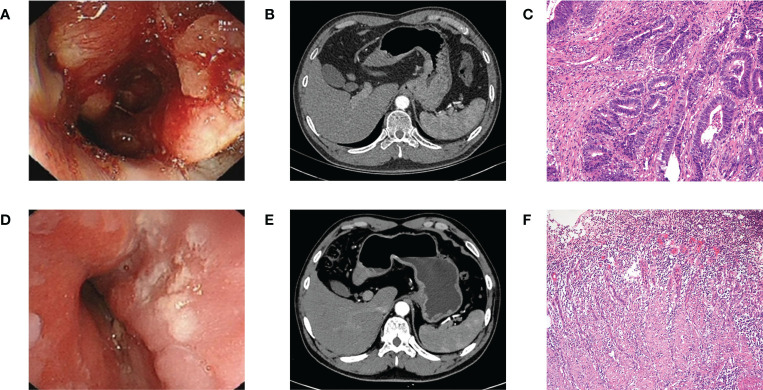
**(A)** Endoscopy before NACT plus tislelizumab. **(B)** CT before NACT plus tislelizumab. **(C)** Pathology before NACT plus tislelizumab. **(D)** Endoscopy after NACT plus tislelizumab. **(E)** CT after NACT plus tislelizumab. **(F)** Pathology after NACT plus tislelizumab. NACT, neoadjuvant chemotherapy.

Patients routinely received two to four cycles of SOX (oxaliplatin, 130 mg/m^2^ intravenously on day 1 every 3 weeks; S-1, 40 mg/m^2^ orally twice daily for 2 weeks every 3 weeks) or FOLFOX (oxaliplatin, 85 mg/m^2^ intravenously on day 1 every 2 weeks, leucovorin, 400 mg/m^2^ intravenously on day 1 every 2 weeks; fluorouracil, 400 mg/m^2^ on day 1 followed by 2400 mg/m^2^ in 46 hours intravenously) NACT regimen or SOX regimen NACT plus tislelizumab (tislelizumab, 200 mg intravenously once in 3 weeks). Patients underwent routine general physical condition and hematological examination before every cycle, and those who received NACT plus tislelizumab underwent additional examination of corticosteroids, thyroid function, and myocardial enzymes, among others. Endoscopic ultrasonography and CT were performed to assess the disease status of the patients after every two to three cycles (**Figure 1**). Further treatment options were determined after multidisciplinary treatment (MDT) discussion. Radical surgery was considered if the effectiveness evaluation revealed a stable disease or partial response with estimated R0 resection. Other systemic treatments were considered if the effectiveness evaluation revealed difficulty achieving R0 resection and progressive disease. Adverse events during neoadjuvant therapy were graded according to the Common Terminology Criteria for Adverse Events (22). Patients with severe adverse effects (grade 3 or higher) were given the necessary medical care.

The methods and timing of surgery were determined after MDT discussion according to tumor size, location, and response to neoadjuvant therapy. Postoperative pathological TNM staging was graded in accordance with the 8th American Joint Committee on Cancer Gastric Cancer Staging Manual. Pathological responses to neoadjuvant therapy were classified according to tumor regression grade (TRG) (6). The Clavien–Dindo grading system was used to evaluate the postoperative complications (23). The adjuvant chemotherapy regimen was determined based on the patient’s general physical condition and pathological reactions.

### Data collection

The data collected were as follows: (1) baseline information of patients including sex, age, tumor location, clinical TNM stage and so on; (2) surgical and postoperative pathological information including operative intervals between neoadjuvant therapy and surgery, surgical methods, intraoperative blood loss, R0 resection rate, number of dissected lymph nodes, TRG, and TNM stage; and (3) postoperative recovery information including postoperative complications, time of the first postoperative fluid intake, and postoperative hospital stay.

### Statistical analysis

The SPSS software program (26.0 version, SPSS Inc, Chicago, IL, USA) was used to conduct statistical analyses. Normally distributed measurement data are described as mean±standard deviation, and non-normally distributed measurement data are described as median and inter quartile range. Categorical variables are presented as frequencies (percentages). The Mann–Whitney test or independent sample t-test was performed to evaluate continuous data, and the Fisher’s exact test or chi-square test was conducted for the comparisons of categorical variables. *P* value<0.05 was set to indicate statistical significance.

## Results

### Baseline data

**Figure 2:**
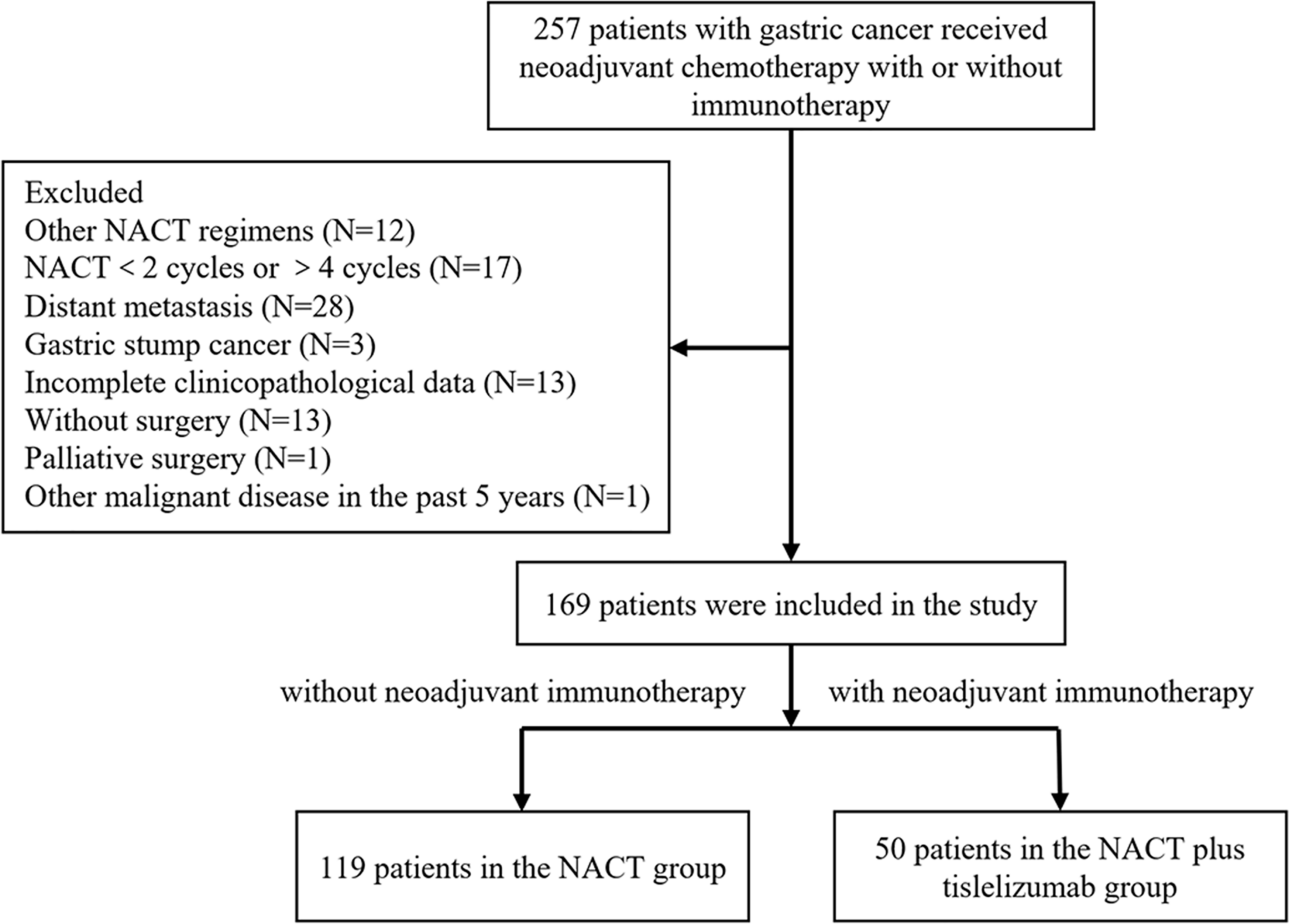
Patients included in this study according to the inclusion and exclusion criteria.

A total of 119 LAGC patients receiving NACT and 50 LAGC patients receiving NACT plus tislelizumab were enrolled in this study. The screening process is shown in **Figure 2**. The NACT group comprised 103 men (86.6%) and 16 women (13.4%). The number of patients with cT 2, 3 and 4 were 9 (7.6%), 45 (37.8%), 65 (54.6%), with cN 0-1 and 2-3 were 80 (67.2%), 39 (32.8%) and in TNM stages II and III were 12 (10.1%) and 107 (89.9%), respectively. The NACT plus tislelizumab group comprised 39 men (78.0%) and 11 women (22.0%). The number of patients with cT 2, 3 and 4 were 2 (4.0%), 20 (40.0%), 28 (56.0%), with cN 0-1 and 2-3 were 34 (68.0%), 16 (32.0%) and in TNM stages II and III were 3 (6.0%) and 47 patients (94.0%), respectively. No significant difference was found between the baseline data of the two groups (**Table 1**).

**Table 1:** Baseline characteristics of patients with locally advanced gastric cancer in the NACT and NACT plus tislelizumab groups.

Variables	Total (n=169)	NACT group (N=119)	NACT plus tislelizumab group (N=50)	*P value*
Gender				*0.166*
Female	27	16(13.4%)	11(22.0%)	
Male	142	103(86.6%)	39(78.0%)	
Age				*0.914*
<65 y	126	89(74.8%)	37(74.0%)	
≥65 y	43	30(25.2%)	13(26.0%)	
BMI		22.5±4.1	22.5±3.0	*0.976*
ASA				*0.364*
1	27	17(14.3%)	10(20.0%)	
2	139	99(83.2%)	40(80.0%)	
3	3	3(2.5%)	0(0%)	
Underlying diseases				*0.378*
No	124	85(71.4%)	39(78.0%)	
Yes	45	34(28.6%)	11(22.0%)	
Preoperative Hb (g/L)		120.0±18.0	115.6±15.6	*0.306*
Preoperative albumin (g/L)		39.1±4.6	37.0±3.6	*0.176*
Tumor location				*0.501*
esophagogastric	64	47(39.5%)	17(34.0%)	
non-esophagogastric	105	72(60.5%)	33(66.0%)	
cT				*0.690*
2	11	9(7.6%)	2(4.0%)	
3	65	45(37.8%)	20(40.0%)	
4	93	65(54.6%)	28(56.0%)	
cN				*0.922*
0-1	114	80(67.2%)	34(68.0%)	
2-3	55	39(32.8%)	16(32.0%)	
cTNM before neoadjuvant therapy				*0.557*
II	15	12(10.1%)	3(6.0%)	
III	154	107(89.9%)	47(94.0%)	

NACT, Neoadjuvant chemotherapy; BMI, Body Mass Index; ASA, American Society of Anesthesiologists.

### Surgical and postoperative pathological information

In the NACT group, 63 (52.9%) and 56 (47.1%) patients underwent laparoscopic surgery and open surgery, respectively. In the NACT plus tislelizumab group, all patients underwent laparoscopic surgery. No significant difference was found in operative time (210.5 ± 70.4 min *vs.* 237.6 ± 68.4 min, *P*=0.732) and intraoperative blood loss (157.8 ± 75.9 ml *vs.* 149.1 ± 92.5 ml, *P*=0.525) between the NACT and NACT plus tislelizumab groups.

The number of dissected lymph nodes showed no significant difference between the NACT and NACT plus tislelizumab groups (24.7 ± 9.3 *vs.* 28.1 ± 10.3, *P*>0.05). In comparison to the NACT plus tislelizumab group, the R0 resection rate (100% *vs.* 89.9%, *P*=0.019) and pathologic complete response rate (26.0% *vs.* 3.4%, *P*<0.001) in the NACT group were significantly lower. TRGs 0, 1, 2, and 3 were reported in 4 (3.4%), 11 (9.2%), 34 (28.6%), and 70 (58.8%) patients in the NACT group and in 14 (28.0%), 8 (16.0%), 11 (22.0%), and 17 (34.0%) patients in the NACT plus tislelizumab group, respectively, with significant differences (*P*<0.001). In the NACT group, 28 (23.5%) and 91 (76.5%) patients were diagnosed as ypT 0-2 and 3-4, 58 (48.7%) and 61 (51.3%) patients as ypN 0-1 and 2-3, respectively; In the NACT plus tislelizumab group, 23 (46.0%) and 27 (54.0%) patients were diagnosed as ypT 0-2 and 3-4, and 37 (74.0%) and 13 (26.0%) patients as ypN 0-1 and 2-3, respectively. Significant difference was found between the two groups (*P*=0.004; *P*=0.003). ypTNM stages 0, I, II, III, and IV were found in 4 (3.4%), 14 (11.8%), 34 (28.6%), 60 (50.4%), 7 patients (5.9%) in the NACT group and in 13 (26.0%), 7 (14.0%), 17 (34.0%), 12 (24.0%), and 1 patient(s) (2.0%) in the NACT plus tislelizumab group, respectively, with significant differences (*P*<0.001) (**Table 2**).

**Table 2:** Baseline characteristics of patients with locally advanced gastric cancer in the NACT and NACT plus tislelizumab groups.

Variables	Total (n=169)	NACT group (N=119)	NACT plus tislelizumab group (N=50)	*P value*
Interval time between neoadjuvant therapy and surgery				*0.389*
<5 wk	83	61 (51.3%)	22 (44.0%)	
≥5 wk	86	58 (48.7%)	28 (56.0%)	
Operation method				*<0.001*
laparoscopic	113	63(52.9%)	50(100%)	
open	56	56(47.1%)	0(0%)	
Resection type				*<0.001*
Proximal	28	27(22.7%)	1(2.0%)	
Distal	34	17(14.3%)	17(34.0%)	
Total	107	75(63.0%)	32(64.0%)	
Operative time (min)		210.5±70.4	237.6±68.4	*0.732*
Intraoperative blood loss (ml)		157.8±75.9	149.1±92.5	*0.609*
Number of lymph node harvested		24.7±9.3	28.1±10.3	*0.195*
Nerve invasion				*0.181*
No	88	58(48.7%)	30(60.0%)	
Yes	81	61(51.3%)	20(40.0%)	
Vascular invasion				*0.307*
No	112	76(63.9%)	36(72.0%)	
Yes	57	43(36.1%)	14(28.0%)	
Margin status				*0.019*
R0	157	107(89.9%)	50(100%)	
R1	12	12(10.1%)	0(0%)	
TRG				*<0.001*
0	18	4(3.4%)	14(28.0%)	
1	19	11(9.2%)	8(16.0%)	
2	45	34(28.6%)	11(22.0%)	
3	87	70(58.8%)	17(34.0%)	
pCR	17	4(3.4%)	13(26.0%)	*<0.001*
ypT				*0.004*
0-2	51	28 (23.5%)	23 (46.0%)	
3-4	118	91 (76.5%)	27 (54.0%)	
ypN				*0.003*
0-1	95	58 (48.7%)	37 (74.0%)	
2-3	74	61 (51.3%)	13 (26.0%)	
ypTNM				*<0.001*
0	17	4(3.4%)	13(26.0%)	
I	21	14(11.8%)	7(14.0%)	
II	51	34(28.6%)	17(34.0%)	
III	72	60(50.4%)	12(24.0%)	
IV	8	7(5.9%)	1(2.0%)	

NACT, Neoadjuvant chemotherapy; TRG, Tumor regression grade; pCR, pathological complete response.

### Complications and short−term outcomes

No statistically significant difference was found in the time of the first postoperative fluid intake and defecation (3.6 ± 2.0 days *vs.* 3.2 ± 2.5 days, *P*=0.456; 5.0 ± 2.5 days *vs.* 4.5 ± 2.4 days, *P*=0.773) between the NACT and NACT plus tislelizumab groups. Postoperative complications occurred in 42 patients (24.9%), with an incidence of 24.4% and 26.0% in the NACT and NACT plus tislelizumab groups, respectively, with no significant difference (*P*=0.823). Postoperative complications mainly included pleural effusion (5.9% *vs.* 8.0%), lung infection (10.1% *vs.* 14.0%), gastroparesis (2.5% *vs.* 2.0%), intestinal obstruction (2.5% *vs.* 4.0%), and anastomosis-related complications (3.4% *vs.* 2.0%) in the NACT and NACT plus tislelizumab groups, with no statistically significant (*P*>0.05). Nine patients in the NACT group had Clavien–Dindo grade 3 or higher complications, including two cases of acute respiratory distress syndrome, three cases of anastomosis-related complications, one case of acute respiratory distress syndrome combined with anastomosis-related complications, one case of abdominal bleeding, one case of gastroparesis, and one case of cerebral infarction, all of which improved after treatment. Twelve patients in the NACT plus tislelizumab group had Clavien–Dindo grade 1-2 complications, including three cases of pleural effusion, five cases of pulmonary infection, one case of pleural effusion plus pulmonary infection, one case of pulmonary infection plus gastroparesis, one case of pneumothorax plus intestinal obstruction and one case of intestinal obstruction plus anastomose-related complications. One patient in the NACT combined with immunotherapy group developed hemophagocytic syndrome (Clavien–Dindo grade 3 or higher complication) and died 27 days after surgery. The postoperative hospital stay was within 12 days in 95 (79.8%) and 44 (89.8%) patients in the NACT and NACT plus tislelizumab groups, with no significant difference (*P*>0.05) (**Table 3**).

**Table 3:** Postoperative complications and recovery of patients with locally advanced gastric cancer in the NACT and NACT plus tislelizumab groups.

Variables	Total (n=169)	NACT group (N=119)	NACT plus tislelizumab group (N=50)	*P value*
Postoperative complications				*0.823*
No	127	90(75.6%)	37(74.0%)	
Yes	42	29(24.4%)	13(26.0%)	
Clavein-dindo classification				*0.245*
Grade 0	127	90(75.6%)	37(74.0%)	
Grade 1-2	32	20(16.8%)	12(24.0%)	
≥Grade 3	10	9(7.6%)	1(2.0%)	
Specific postoperative complications				
Pleural effusion	11	7(5.9%)	4(8.0%)	*0.734*
Pulmonary infection	19	12(10.1%)	7(14.0%)	*0.462*
Pneumothorax	3	2(1.7%)	1(2.0%)	*1*
ARDS	3	3(2.5%)	0(0%)	*0.556*
Gastroparesis	4	3(2.5%)	1(2.0%)	*1*
Intestinal obstruction	5	3(2.5%)	2(4.0%)	*0.633*
Anastomose-related complications	5	4(3.4%)	1(2.0%)	*1*
Intra-abdominal hemorrhage	1	1(0.8%)	0(0%)	*1*
Wound infection	2	2(1.7%)	0(0%)	*1*
Cerebral infarction	1	1(0.8%)	0(0%)	*1*
Hemophagocytic syndrome	1	0(0%)	1(2.0%)	*0.296*
Time of the first postoperative fluid intake (d)		3.6±2.0	3.2±2.5	*0.456*
Time of the first defecation (d)		5.0±2.5	4.5±2.4	*0.773*
Postoperative hospital stay				*0.120*
≤12 d	139	95(79.8%)	44(89.8%)	
>12 d	29	24(20.2%)	5(10.2%)	
30-day mortality (n)	1	0(0%)	1(2.0%)	*0.296*

NACT, Neoadjuvant chemotherapy; ARDS, acute respiratory distress syndrome.

### Subgroup analysis based on tumor location

A subgroup analysis was performed for patients with esophagogastric junction carcinoma and non-esophagogastric junction carcinoma (**Table 4**). Among those with esophagogastric junction carcinoma, 13 (27.7%) had TRGs of 0–2 and 34 (72.3%) had a TRG of 3 in the NACT group, while 13 (76.5%) had TRGs of 0–2 and 4 (23.5%) had a TRG of 3 in the NACT plus tislelizumab group, and the difference was statistically significant (*P*<0.001). The proportion of patients with ypT (stages 3–4: 87.2% *vs.* 41.2%, *P*<0.001), ypN (stages 2–3: 46.8% *vs.* 5.9%, *P*=0.003), and ypTNM stages (stages III-IV: 87.2% *vs.* 41.2%, *P*<0.001) in the NACT group was significantly higher than in the NACT plus tislelizumab group. Among those with non-esophagogastric junction carcinoma, the NACT and NACT plus tislelizumab groups showed no significant difference in TRG (TRG 3: 50.0% *vs.* 39.4%, *P*=0.312), ypT stage (stages 3–4: 69.4% *vs.* 60.6%, *P*=0.372), ypN stage (stages 2–3: 54.2% *vs.* 36.4%, *P*=0.090), and ypTNM stage (stages III-IV: 54.2% *vs.* 36.4%, *P*=0.090).

**Table 4:** The subgroup analysis of patients with locally advanced gastric cancer in the NACT and NACT plus tislelizumab groups based on tumor location.

Variables	Esophagogastric junction		*P value*	Non-esophagogastric junction		*P value*
	NACT group (N=47)	NACT plus tislelizumab group (N=17)		NACT group (N=72)	NACT plus tislelizumab group (N=33)	
Nerve invasion			*0.206*			*0.471*
No	22(46.8%)	11(64.7%)		36(50.0%)	19(57.6%)	
Yes	25(53.2%)	6(35.3%)		36(50.0%)	14(42.4%)	
Vascular invasion			*0.158*			*0.782*
No	30(63.8%)	14(82.4%)		46(63.9%)	22(66.7%)	
Yes	17(36.2%)	3(17.6%)		26(36.1%)	11(33.3%)	
Margin status			*0.313*			*0.095*
R0	42(89.4%)	17(100%)		65(90.3%)	33(100%)	
R1	5(10.6%)	0(0%)		7(9.7%)	0(0%)	
TRG			*<0.001*			*0.312*
0-2	13(27.7%)	13(76.5%)		36(50.0%)	20(60.6%)	
3	34(72.3%)	4(23.5%)		36(50.0%)	13(39.4%)	
ypT			*<0.001*			*0.372*
T0-T2	6(12.8%)	10(58.8%)		22(30.6%)	13(39.4%)	
T3-T4	41(87.2%)	7(41.2%)		50(69.4%)	20(60.6%)	
ypN			*0.003*			*0.090*
N0-N1	25(53.2%)	16(94.1%)		33(45.8%)	21(63.6%)	
N2-N3	22(46.8%)	1(5.9%)		39(54.2%)	12(36.4%)	
ypTNM			*<0.001*			*0.090*
0-II	19(40.4%)	16(94.1%)		33(45.8%)	21(63.6%)	
III-IV	28(59.6%)	1(5.9%)		39(54.2%)	12(36.4%)	

NACT, Neoadjuvant chemotherapy; TRG, Tumor regression grade.

## Discussion

In this study, compared with NACT alone, NACT combined with tislelizumab significantly improved the R0 resection rate, pCR rate and tumor regression response of LAGC, particularly in esophagogastric junction carcinoma, and did not increase the incidence of postoperative complications or length of hospital stay. Based on the previous single-arm studies, this study further demonstrated that NACT combined with tislelizumab has better short-term efficacy and comparable safety than NACT alone.

**Figure 3:**
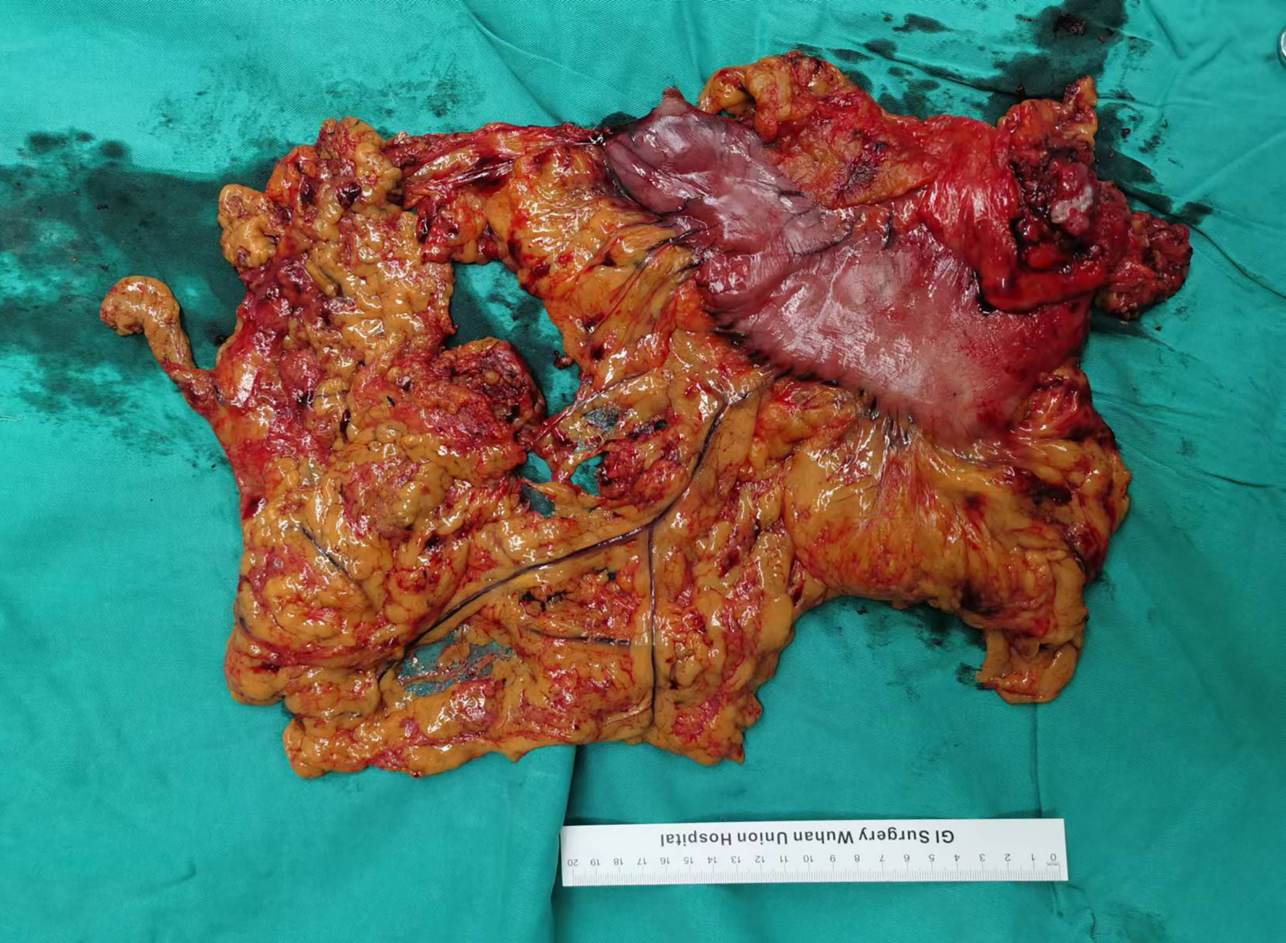
Surgical specimens after NACT plus tislelizumab followed by radical gastrectomy. NACT, neoadjuvant chemotherapy.

The CLASS01 study confirmed that laparoscopic surgery for patients with gastric cancer has a better short-term prognosis than open surgery and does not increase the risk of postoperative recurrence and metastasis (24). Therefore, minimally invasive surgery has been broadly used for gastric cancer in recent years, and the proportion of patients with gastric cancer undergoing laparoscopic surgery in our center has also annually increased. A previous study suggests that immunotherapy may lead to formation of dense fibrosis of the tissue near the lesion in non-small cell lung cancer patients, making thoracoscopic surgery challenging (25, 26). In this study, no serious adhesions or edema around the tumor was observed intraoperatively in patients with LAGC treated with NACT plus immunotherapy, and no significant bleeding or exudation occurred during the separation of the adhesions (**Figure 3**). Operation time and intraoperative blood loss, which are important measures of surgical difficulty, did not increase in patients treated with NACT plus immunotherapy than those treated with NACT alone. The number of dissected lymph nodes and R0 resection rate are important indicators of surgical quality. A meta-analysis including 13 prospective studies showed that the R0 resection rate in LAGC patients treated with NACT plus immunotherapy was 97%, which was statistically higher than that of patients receiving NACT (19). In this study, the number of dissected lymph nodes in patients treated with NACT plus immunotherapy was comparable with that in patients treated with NACT, and all were greater than 15. Based on the aforementioned evidence, NACT plus immunotherapy for LAGC can significantly improve the quality of surgery without increasing the surgical difficulty.

Postoperative complications are an important aspect of assessing the safety of NACT plus immunotherapy. Lin et al. enrolled 200 gastric cancer patients with serosal invasion receiving neoadjuvant therapy, of whom 72 patients received SOX, 95 patients received paclitaxel plus S-1, and 33 patients received camrelizumab and paclitaxel plus S-1 neoadjuvant therapy, showing no statistically difference in the incidence of postoperative complications (20). Similar results were obtained in the present study in patients with LAGC without serosal invasion. Postoperative complications of patients treated with NACT plus immunotherapy mainly included pulmonary infection, pleural effusion, anastomotic leakage, and gastroparesis. The incidence of specific postoperative complications between the two groups was similar in this study, but a series of studies including this one showed that the incidence could be 8%–13.3% for pleural effusion and 14.0%–26.7% for pulmonary infection (20, 27). Therefore, postoperative CT examinations should be performed to detect chest-related complications. In pulmonary infections, distinguishing between bacterial and immune-associated pneumonia is important. Notably, one case of mortality due to severe hemophagocytic syndrome in the NACT plus immunotherapy group suggests that patients should be closely monitored for hematologic indicators, and any abnormalities should be promptly addressed. In addition, NACT plus immunotherapy did not slow the postoperative recovery of patients with LAGC. Overall, NACT combined with immunotherapy is safe and feasible.

Tumor pathological responses to neoadjuvant therapy can accurately reflect treatment efficacy and assess patient prognosis. pCR is a good indicator of the efficacy of neoadjuvant therapy and is an independent predictor of OS, postoperative recurrence and metastasis in gastric cancer patients. The risk of recurrence or death was reduced by half in patients with LAGC with pCR after neoadjuvant therapy (28–30). Previous studies showed that the average pCR rate of patients with LAGC receiving NACT alone is 6.7%, whereas that of patients with LAGC receiving NACT plus immunotherapy can reach 19.4%–33.6% (17, 20, 27, 28). The pCR rate of patients who received NACT plus immunotherapy in this study was 26.0%, and 66.0% of patients showed significant regression at the primary site (TRG 0–2), which was generally consistent with previous studies and significantly higher than that of patients treated with NACT. This may be because chemotherapy can regulate the immune status of the tumor microenvironment and promote the release of cryptic tumor antigens to achieve the synergistic effect of immunotherapy and chemotherapy (31, 32). NACT plus immunotherapy for patients with LAGC showed a significant decrease in pathological TNM stage, but whether this ultimately translates into a long-term survival benefit remains to be further explored. Tumors located at different sites had significantly different biological characteristics. For esophagogastric junction carcinoma, NACT plus immunotherapy was more effective regardless of the primary site or metastatic lymph nodes. By contrast, no significant difference was found in the regression response of non-esophagogastric junction carcinomas to different neoadjuvant therapies. In addition, recent studies have shown that Helicobacter pylori infection can inhibit the proliferation and antitumor effects of CD8+ T cells, promote the differentiation of naive T cells to Tregs, and regulate the expression of inflammatory factors; thus, affecting the tumor immune microenvironment, suppressing the host immune response, and reducing the efficacy of immunotherapy for gastric cancer (33–35). Therefore, attention should be given to tumor location and H. pylori infection in patients with LAGC before neoadjuvant immunotherapy.

This study has several limitations. Firstly, given the retrospective nature of this study over a long-term period, selection bias and changes in treatment could not be prevented, and some patients were not screened for H. pylori infection. Secondly, the sample size in this study was relatively small, which may make it difficult to further explore and validate the results. Third, NACT regimens and operation methods were not standardized and may bias results. In addition, NACT plus immunotherapy is a newly emerging treatment, and long-term prognosis data are lacking. Further validation of these results is required in a large-scale prospective study.

## Conclusions

Compared with SOX or FOLFOX NACT regimen, NACT plus tislelizumab can improve surgical quality without increasing the difficulty of radical gastrectomy. In LAGC, particularly esophagogastric junction carcinoma, NACT plus tislelizumab can significantly improve the R0 resection rate, pCR rate, tumor regression response, and downstaging rate without increasing the rate of perioperative complications. NACT combined with tislelizumab has better short-term efficacy and comparable safety than NACT alone, and has a promising application prospect in the treatment of LAGC. However, its long-term efficacy remains to be investigated.

## Data availability statement

The original contributions presented in the study are included in the article/supplementary material. Further inquiries can be directed to the corresponding author.

## Ethics statement

The studies involving human participants were reviewed and approved by the Institutional Review Board of the Union Hospital of Tongji Medical College. The patients/participants provided their written informed consent to participate in this study. Written informed consent was obtained from the individual(s) for the publication of any potentially identifiable images or data included in this article.

## Author contributions

KT and PZ were in charge of the study concepts and design. QJ, WL, CZ, MY, JF and PZ performed data acquisition, analysis or interpretation. QJ and WL drafted the manuscript and made the figures. Critical revision of the manuscript for important intellectual content: XZ, CZ, YD, LZ, YY, KT and PZ revised the manuscript. All authors contributed to the article and approved the submitted version.
