# Supplementary material for: Safety and efficacy of tislelizumab plus chemotherapy versus chemotherapy alone as neoadjuvant treatment for patients with locally advanced gastric cancer: real-world experience with a consecutive patient cohort
Source: Front Immunol. 2023 May 4;14:1122121. doi: 10.3389/fimmu.2023.1122121 (PMC10195027; doi:10.3389/fimmu.2023.1122121)
Supplement: Supplementary file 1 [file Table_1.docx]

Supplementary table 1 Baseline, surgical and short-term prognosis characteristics of patients with LAGC in the FOLFOX and SOX groups

| Variables | | Total (n=119) | FOLFOX group(n=73) | SOX group(n=46) | χ^2^/t | *P value* |
| --- | --- | --- | --- | --- | --- | --- |
| Gender | |  |  |  | 0.428 | *0.513* |
|  | Female | 16 | 11(15.1%) | 5(10.9%) |  |  |
|  | Male | 103 | 62(84.9%) | 41(89.1%) |  |  |
| Age | |  |  |  | 0.031 | *0.861* |
|  | <65 y | 89 | 55(75.3%) | 34(73.9%) |  |  |
|  | ≥65 y | 30 | 18(24.7%) | 12(26.1%) |  |  |
| BMI | |  | 22.6±4.6 | 22.5±3.2 | 0.064 | *0.949* |
| ASA | |  |  |  | 0.095 | *0.759* |
|  | 1 | 17 | 11(15.1%) | 6(13.0%) |  |  |
|  | 2-3 | 102 | 62(84.9%) | 40(87.0%) |  |  |
| Underlying diseases | |  |  |  | 1.715 | *0.19* |
|  | No | 85 | 49(67.1%) | 36(78.3%) |  |  |
|  | Yes | 34 | 24(32.9%) | 10(21.7%) |  |  |
| Tumor location | |  |  |  | 0.004 | *0.948* |
|  | esophagogastric | 47 | 29(39.7%) | 18(39.1%) |  |  |
|  | non-esophagogastric | 72 | 44(60.3%) | 28(60.9%) |  |  |
| cTNM before neoadjuvant therapy | |  |  |  | 0.051 | *0.821* |
|  | II | 12 | 7(9.6%) | 5(10.9%) |  |  |
|  | III | 107 | 66(90.4%) | 41(89.1%) |  |  |
| Operation method | |  |  |  | 0.018 | *0.894* |
|  | laparoscopic | 63 | 39(53.4%) | 24(52.2%) |  |  |
|  | open | 56 | 34(46.6%) | 22(47.8%) |  |  |
| Resection type | |  |  |  | 3.890 | *0.143* |
|  | Proximal | 27 | 14(19.2%) | 13(28.3%) |  |  |
|  | Distal | 17 | 8(11.0%) | 9(19.6%) |  |  |
|  | Total | 75 | 51(69.9%) | 24(52.2%) |  |  |
| Operative time (min) | |  | 213.6±73.3 | 203.1±64.6 | 0.795 | *0.428* |
| Intraoperative blood loss (ml) | |  | 163.7±84.3 | 148.5±59.9 | 1.066 | *0.289* |
| Postoperative complications | |  |  |  | 0.120 | *0.729* |
|  | No | 90 | 56(76.7%) | 34(73.9%) |  |  |
|  | Yes | 29 | 17(23.3%) | 12(26.1%) |  |  |
| Time of the first postoperative fluid intake (d) | |  | 3.7±2.2 | 3.6±1.7 | 0.265 | *0.791* |
| Time of the first defecation (d) | |  | 5.1±2.6 | 4.9±1.9 | 0.329 | *0.743* |
| Postoperative hospital stay | |  |  |  | 0.017 | *0.896* |
|  | ≤12 d | 95 | 58(79.5%) | 37(80.4%) |  |  |
|  | >12 d | 24 | 15(20.5%) | 9(19.6%) |  |  |
