# Supplementary material for: Safety and efficacy of tislelizumab plus chemotherapy versus chemotherapy alone as neoadjuvant treatment for patients with locally advanced gastric cancer: real-world experience with a consecutive patient cohort
Source: Front Immunol. 2023 May 4;14:1122121. doi: 10.3389/fimmu.2023.1122121 (PMC10195027; doi:10.3389/fimmu.2023.1122121)
Supplement: Supplementary file 2 [file Table_2.docx]

Supplementary table 2 Post-operative pathological characteristics of patients with LAGC in the FOLFOX and SOX groups

| Variables | | Total (n=119) | FOLFOX group(n=73) | SOX group(n=46) | χ^2^/t | *P value* |
| --- | --- | --- | --- | --- | --- | --- |
| Number of lymph node harvested | |  | 24.5±9.1 | 25.2±9.7 | 0.397 | *0.692* |
| Nerve invasion | |  |  |  | 0.354 | *0.552* |
|  | No | 58 | 34(46.6%) | 24(52.2%) |  |  |
|  | Yes | 61 | 39(53.4%) | 22(47.8%) |  |  |
| Vascular invasion | |  |  |  | 1.056 | *0.304* |
|  | No | 76 | 44(60.3%) | 32(69.9%) |  |  |
|  | Yes | 43 | 29(39.7%) | 14(30.4%) |  |  |
| Margin status | |  |  |  | 1.049 | *0.306* |
|  | R0 | 107 | 64(87.7%) | 43(93.5%) |  |  |
|  | R1 | 12 | 9(12.3%) | 3(6.5%) |  |  |
| TRG | |  |  |  | 0.620 | *0.431* |
|  | 0-2 | 49 | 28(38.4%) | 21(45.7%) |  |  |
|  | 3 | 70 | 45(61.6%) | 25(54.3%) |  |  |
| ypT | |  |  |  | 0.273 | *0.602* |
|  | T0-T2 | 28 | 16(21.9%) | 12(28.1%) |  |  |
|  | T3-T4 | 91 | 57(78.1%) | 34(73.9%) |  |  |
| ypN | |  |  |  | 0.944 | *0.331* |
|  | N0-N1 | 58 | 33(45.2%) | 25(54.3%) |  |  |
|  | N2-N3 | 61 | 40(54.8%) | 21(45.7%) |  |  |
| ypTNM | |  |  |  | 0.116 | *0.733* |
|  | 0-II | 52 | 31(42.5%) | 21(45.7%) |  |  |
|  | III-IV | 67 | 42(57.5%) | 25(54.3%) |  |  |
