# Supplementary material for: Safety and efficacy of tislelizumab plus chemotherapy versus chemotherapy alone as neoadjuvant treatment for patients with locally advanced gastric cancer: real-world experience with a consecutive patient cohort
Source: Front Immunol. 2023 May 4;14:1122121. doi: 10.3389/fimmu.2023.1122121 (PMC10195027; doi:10.3389/fimmu.2023.1122121)
Supplement: Supplementary file 3 [file Table_3.docx]

Supplementary table 3 Baseline, surgical and short-term prognosis characteristics of patients with LAGC in the laparoscopic and open surgery groups

| Variables | | Total (n=119) | laparoscopic surgery group(n=63) | open surgery group(n=56) | χ^2^/t | *P value* |
| --- | --- | --- | --- | --- | --- | --- |
| Gender | |  |  |  | 1.854 | *0.173* |
|  | Female | 16 | 11(17.5%) | 5(8.9%) |  |  |
|  | Male | 103 | 52(82.5%) | 51(91.1%) |  |  |
| Age |  |  |  |  | 0.002 | *0.960* |
|  | <65 y | 89 | 47(74.6%) | 42(75.0%) |  |  |
|  | ≥65 y | 30 | 16(25.4%) | 14(25.0%) |  |  |
| BMI | |  | 22.8±2.5 | 22.2±4.7 | 0.646 | *0.520* |
| ASA | |  |  |  | 0.275 | *0.600* |
|  | 1 | 17 | 10(15.9%) | 7(12.5%) |  |  |
|  | 2-3 | 102 | 53(84.1%) | 49(87.5%) |  |  |
| Underlying diseases | |  |  |  | 0.661 | *0.416* |
|  | No | 85 | 43(68.3%) | 42(75.0%) |  |  |
|  | Yes | 34 | 20(31.7%) | 14(25.0%) |  |  |
| Tumor location | |  |  |  | 0.500 | *0.479* |
|  | esophagogastric | 47 | 23(36.5%) | 24(42.9%) |  |  |
|  | non-esophagogastric | 72 | 40(63.5%) | 32(57.1%) |  |  |
| cTNM before neoadjuvant therapy | |  |  |  | 0.681 | *0.409* |
|  | II | 12 | 5(7.9%) | 7(12.5%) |  |  |
|  | III | 107 | 58(92.1%) | 49(87.5%) |  |  |
| NACT regimen | |  |  |  | 0.018 | *0.894* |
|  | FOLFOX | 73 | 39(61.9%) | 34(60.7%) |  |  |
|  | SOX | 46 | 24(38.1%) | 22(39.3%) |  |  |
| Resection type | |  |  |  | 0.490 | *0.783* |
|  | Proximal | 27 | 13(20.6%) | 14(25.0%) |  |  |
|  | Distal | 17 | 10(15.9%) | 7(12.5%) |  |  |
|  | Total | 75 | 40(63.5%) | 35(62.5%) |  |  |
| Operative time (min) | |  | 214.0±73.8 | 206.7±66.9 | 0.561 | *0.576* |
| Intraoperative blood loss (ml) | |  | 164.9±88.5 | 149.8±58.3 | 1.084 | *0.280* |
| Postoperative complications | |  |  |  | 2.057 | *0.151* |
|  | No | 90 | 51(81.0%) | 39(69.6%) |  |  |
|  | Yes | 29 | 12(19.0%) | 17(30.4%) |  |  |
| Time of the first postoperative fluid intake (d) | |  | 3.7±2.4 | 3.5±1.5 | 0.342 | *0.733* |
| Time of the first defecation (d) | |  | 4.9±2.3 | 5.1±2.1 | 0.364 | *0.717* |
| Postoperative hospital stay | |  |  |  | 0.610 | *0.435* |
|  | ≤12 d | 95 | 52(82.5%) | 43(76.8%) |  |  |
|  | >12 d | 24 | 11(17.5%) | 13(23.2%) |  |  |
