# Supplementary material for: Safety and efficacy of tislelizumab plus chemotherapy versus chemotherapy alone as neoadjuvant treatment for patients with locally advanced gastric cancer: real-world experience with a consecutive patient cohort
Source: Front Immunol. 2023 May 4;14:1122121. doi: 10.3389/fimmu.2023.1122121 (PMC10195027; doi:10.3389/fimmu.2023.1122121)
Supplement: Supplementary file 4 [file Table_4.docx]

Supplementary table 4 Post-operative pathological characteristics of patients with LAGC in the laparoscopic and open surgery groups

| Variables | | Total (n=119) | laparoscopic surgery group(n=63) | open surgery group(n=56) | χ^2^/t | *P value* |
| --- | --- | --- | --- | --- | --- | --- |
| Number of lymph node harvested | |  | 25.2±9.2 | 24.2±9.6 | 0.598 | *0.551* |
| Nerve invasion | |  |  |  | 0.711 | *0.399* |
|  | No | 58 | 33(52.4%) | 25(44.6%) |  |  |
|  | Yes | 61 | 30(47.6%) | 31(55.4%) |  |  |
| Vascular invasion | |  |  |  | 0.730 | *0.393* |
|  | No | 76 | 38(60.3%) | 38(67.9%) |  |  |
|  | Yes | 43 | 25(39.7%) | 18(32.1%) |  |  |
| Margin status | |  |  |  | 1.009 | *0.315* |
|  | R0 | 107 | 55(87.3%) | 52(92.9%) |  |  |
|  | R1 | 12 | 8(12.7%) | 4(7.1%) |  |  |
| TRG | |  |  |  | 0.156 | *0.693* |
|  | 0-2 | 49 | 27(42.9%) | 22(39.3%) |  |  |
|  | 3 | 70 | 36(57.1%) | 34(60.7%) |  |  |
| ypT | |  |  |  | 0.623 | *0.430* |
|  | T0-T2 | 28 | 13(20.6%) | 15(26.8%) |  |  |
|  | T3-T4 | 91 | 50(79.4%) | 41(73.2%) |  |  |
| ypN | |  |  |  | 0.012 | *0.914* |
|  | N0-N1 | 58 | 31(49.2%) | 27(48.2%) |  |  |
|  | N2-N3 | 61 | 32(50.8%) | 29(51.8%) |  |  |
| ypTNM | |  |  |  | 0.038 | *0.845* |
|  | 0-II | 52 | 27(42.9%) | 25(44.6%) |  |  |
|  | III-IV | 67 | 36(57.1%) | 31(55.4%) |  |  |
